# REPLY TO THE AUTHORS: Re: Simplified Fournier's gangrene severe index score (SFGSI)

**DOI:** 10.1590/S1677-5538.IBJU.2017.0535.1

**Published:** 2019

**Authors:** Carlos Eugênio Lira Tenório, Salvador Vilar Correia Lima, Amanda Vasconcelos de Albuquerque, Mariana Pauferro Cavalcanti, Flávio Teles

**Affiliations:** 1 Serviço de Urologia do Hospital das Clínicas, Departamento de Cirurgia do Centro de Ciências da Saúde da Universidade Federal de Pernambuco, UFPE, Recife, PE, Brasil;; 2 Faculdade de Medicina da Universidade Estadual de Ciências da Saúde de Alagoas (UNCISAL), Maceió, AL, Brasil;; 3 Núcleo de Cirurgia Experimental, Programa de Pós-Graduação em Cirurgia, Departamento de Cirurgia, Centro de Ciências da Saúde, Universidade Federal de Pernambuco, UFPE, Recife, PE, Brasil

*To the editor,*

We thank Yasri and Wiwanitkit (1) for their knowledgeable comments on our study. One concern was that the simplified form of Fournier's Gangrene Severe Index Score (SFGSI) could not be used immediately since it needs laboratory variables, sometimes not available at admission. Indeed, “few minutes after” could be more appropriated than “immediately” in this case. However, in current emergency practice, hemoglobin, potassium and creatinine can be performed quickly with a small blood sample even in public hospitals of low-income countries like Brazil. Another concern was that hemoglobin “is affected by other possible concomitant disorders”. In agreement with previous evidences (2, 3), our study demonstrated an association between hematocrit and mortality in Fournier's Gangrene using a logistic regression model, in which we also inserted other variables that were associated with death in univariate analysis. The use of scores has the advantage of scape from random associations of single variables and can have greater predictive power. Therefore, the strongest point of our study was not the single association of hematocrit with mortality, but the demonstration that when hematocrit, potassium and creatinine were tested together, as part of the SFGSI, there was the largest independent association with mortality (4).

We know that correlation does not imply causation and it is important to establish whether our findings hold in other centers.

In summary, we strongly believe that our results support previous evidence and are a useful addition to our toolbox in predicting mortality in patients with Fournier's Gangrene.
